# RB loss sensitizes triple-negative breast cancer to apoptosis induced by cellular stress

**DOI:** 10.1038/s41420-025-02864-4

**Published:** 2025-11-24

**Authors:** Agnieszka K. Witkiewicz, Subrahmanya Anirudh Kaligotla Venkata, Erik S. Knudsen, Vishnu Kumarasamy

**Affiliations:** Department of Molecular and Cellular Biology, Roswell Park Comprehensive Cancer Center, Buffalo, NY USA

**Keywords:** Breast cancer, High-throughput screening

## Abstract

Functional loss of *RB1* is a common genetic alteration in triple-negative breast cancer (TNBC) and is associated with poor response to targeted therapies, including CDK4/6 inhibitors. In this study, we perform an unbiased drug screen and identify that co-targeting distinct cell cycle processes such as DNA repair and mitosis induce synthetic lethality selectively in RB-deficient models. While RB loss promotes replication stress and mitotic dysregulation, the selective lethality observed with these combinations arises from an alternate mechanism. Under RB-deficient conditions, cells undergo rapid apoptosis in response to cellular stress induced by cell cycle inhibition. This pro-apoptotic response is further augmented by using a pharmacological agent, birinapant that targets XIAP, which is an endogenous inhibitor of the apoptotic pathway. Birinapant in combination with CHK1 or AURKA inhibitors results in selective cell killing in RB-deficient TNBC models and yields durable disease control via apoptosis in vivo. In conclusion, RB loss in TNBC displays an enhanced vulnerability to pro-apoptotic signaling that can enable the effective implementation of new targeted therapeutic strategies.

## Introduction

Triple-negative breast cancer (TNBC) is an aggressive subtype that accounts for 15–20% of all breast cancer cases [1, 2]. TNBC is associated with poor prognosis and the worst survival outcomes of all breast cancer subtypes, with a 5-year survival rate of 12%. This is largely due to lack of effective treatment options. Currently, chemotherapy remains the standard of care, but its clinical success is often limited by high recurrence rates and adverse toxicity [3, 4]. These challenges underscore the urgent need for novel therapeutic strategies to improve patient outcomes.

Recent advances in molecular targeted therapies have provided promising clinical benefits for a subset of TNBC patients. For example, PARP inhibitors have shown clinical efficacy, but it is limited to patients with BRCA1 mutations [5–7]. Beyond this, other targeted therapies that are being investigated include inhibition of the PI3K/mTOR signaling pathway and the cell cycle regulatory elements CDK4 and 6 kinases, which both play critical roles in cancer cell proliferation [8–10]. The development of CDK4/6 inhibitors has been a major clinical breakthrough in estrogen receptor-positive (ER + ) breast cancer patients, significantly improving overall survival when combined with endocrine therapy [11–14]. However, TNBC lacks expression of the hormone receptors and HER2, thereby rendering it refractory to such treatment options [15]. As a result, preclinical studies are now exploring the combination of CDK4/6 and PI3K/mTOR inhibitors as a potential therapeutic strategy for TNBC [10, 16]. Mechanistically, this approach relies on activation of a tumor suppressor protein, RB, which acts as a checkpoint regulator during the G1-S phase transition of the cell cycle [17, 18]. RB exerts inhibitory effects on E2F transcriptional factors, which is relieved upon phosphorylation by CDK4 and 6 kinases [19, 20]. Thus, CDK4/6 inhibitors block RB phosphorylation and suppress the E2F target genes that are essential for cell cycle progression, thereby inducing cell cycle arrest [21, 22].

A major challenge in utilizing CDK4/6 inhibitors as a therapeutic strategy in TNBC stems from the frequent functional loss of the RB protein, the primary substrate for CDK4/6 kinases [23, 24]. RB loss is a key mechanism of resistance to these inhibitors and significantly impacts the clinical outcomes and patient survival [17, 25, 26]. Therefore, the therapeutic limitations in TNBC arise from tumor heterogeneity, which drives resistance and necessitates more precise approaches depending on the genetic background. As a tumor suppressor, RB loss disrupts cell cycle checkpoints, leading to aberrant DNA replication and replication stress. This vulnerability has been therapeutically exploited using DNA-damaging chemotherapeutic agents, which display enhanced efficacy in RB-deficient tumors [23, 27]. There are emerging evidences highlighting the non-canonical functions of RB beyond cell cycle regulation, including but not limited to chromatin organization, autophagy, and impacting the tumor microenvironment, which are all involved in tumor suppression [28]. Therefore, understanding RB-regulated pathways to which tumor cells become addicted following RB loss may uncover novel therapeutic opportunities. In this study, we uncovered a distinct phenotype in which RB loss makes tumor cells vulnerable to apoptotic pathway targeting, offering a new avenue for precision therapy in RB-deficient TNBC.

## Results

### RB loss drives therapeutic vulnerability in TNBC

**Fig. 1:**
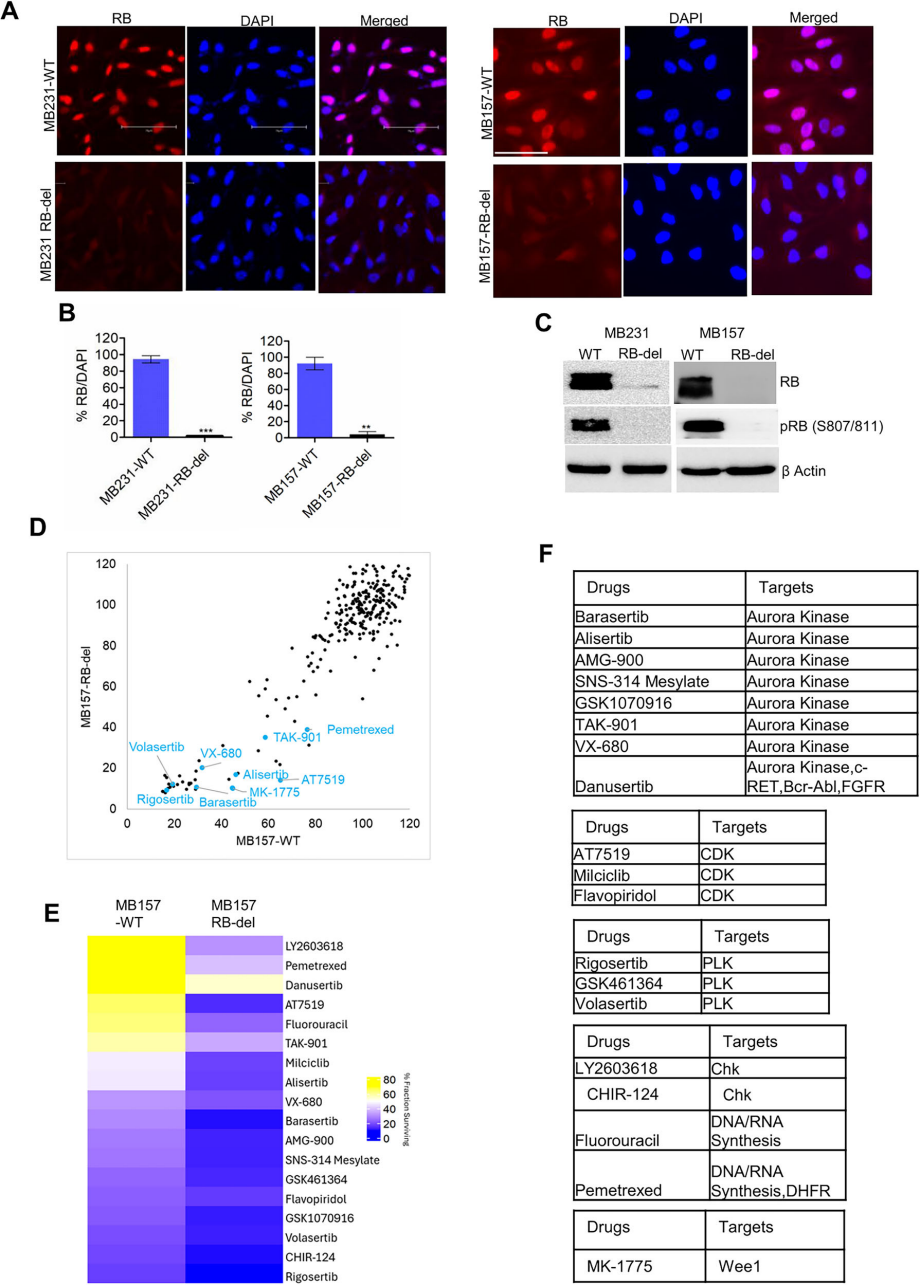
Drug screening analysis in TNBC cell lines that harbor genetic RB-deletion. **A** Immunofluorescence staining to examine the expression of RB in MB231-WT, MB231-RB-del, MB157-WT and MB157-RB-del cell lines. The cells were counterstained with the nuclear dye DAPI. The scale bar represents 75 µm. **B** Column graphs illustrating the fraction of RB-positive cells from the MB231-WT, MB231-RB-del, MB157-WT and MB157-RB-del cells based on immunofluorescence analysis. Error bars represent mean and SD from three independent experiments. ***p < 0.0001 as determined by student t test. **C** Western blot analysis to examine RB and pRB expression from the MB231-WT, MB231-RB-del, MB157-WT and MB157-RB-del cells. **D** Correlation plot comparing the % fraction of live cells between MB157-WT (X-axis) and MB157-RB-del (Y-axis) cells following exposure to the drug library as determined by the CellTiter Glo (CTG) assay. **E** Heat map depicting the differential response to the indicated drugs in MB157-WT and RB-del cells based on % fraction survival as determined by the CTG assay. **F** List of drugs and their associated targets that were selectively potent in MB157-RB-del cells.

Inactivation of RB by CDK4/6 and CDK2 kinases is a critical step in cell cycle progression [29]. Our prior studies demonstrated that in RB-proficient TNBC models, MB231 and MB157, pharmacological inhibition of CDK4/6 and CDK2, respectively, is sufficient to activate RB and arrest cell cycle progression, confirming its functional role in mediating therapeutic responses [23, 30]. Therefore, to investigate the therapeutic consequences of RB loss, we stably deleted RB using CRISPR/Cas9 and generated MB231-RB-del and MB157-RB-del cell lines. To validate RB loss in both models, its expression was evaluated by immunofluorescence, which revealed that in both MB231-RB-del and MB157-RB-del cell lines RB expression was significantly suppressed as compared to their wildtype counterparts (MB231-WT and MB157-WT) (Fig. 1A, B). Biochemical analysis further confirmed that the expression of RB and phospho-RB were reduced in the RB-del, MB231, and MB157 cells (Fig. 1C). To assess the impact of RB loss on therapeutic outcomes, an unbiased drug screening analysis was performed in the MB157-WT and MB157-RB-del cell lines. The cells were exposed to a drug library comprising different targeted and chemotherapeutic agents, and the responses to the individual drugs were determined based on the impact on cell viability as determined by CTG assay. A correlation analysis comparing the cell viability between wildtype and RB-deleted cell lines revealed that RB loss renders the cells to be more vulnerable to different therapeutic agents as observed by reduced cell viability (Fig. 1D, E). These drugs were categorized into functional families with shared molecular targets, which mainly included Aurora kinase (AURK) inhibitors, polo-like-kinase (PLK) inhibitors, CHK kinase inhibitors, DNA replication inhibitors and CDK inhibitors (Fig. 1F). Given that these targets are known to interfere with different cell cycle processes that are regulated by RB, our findings suggest that RB deficiency enhances sensitivity to these inhibitors creating potential therapeutic opportunities.

**Fig. 2:**
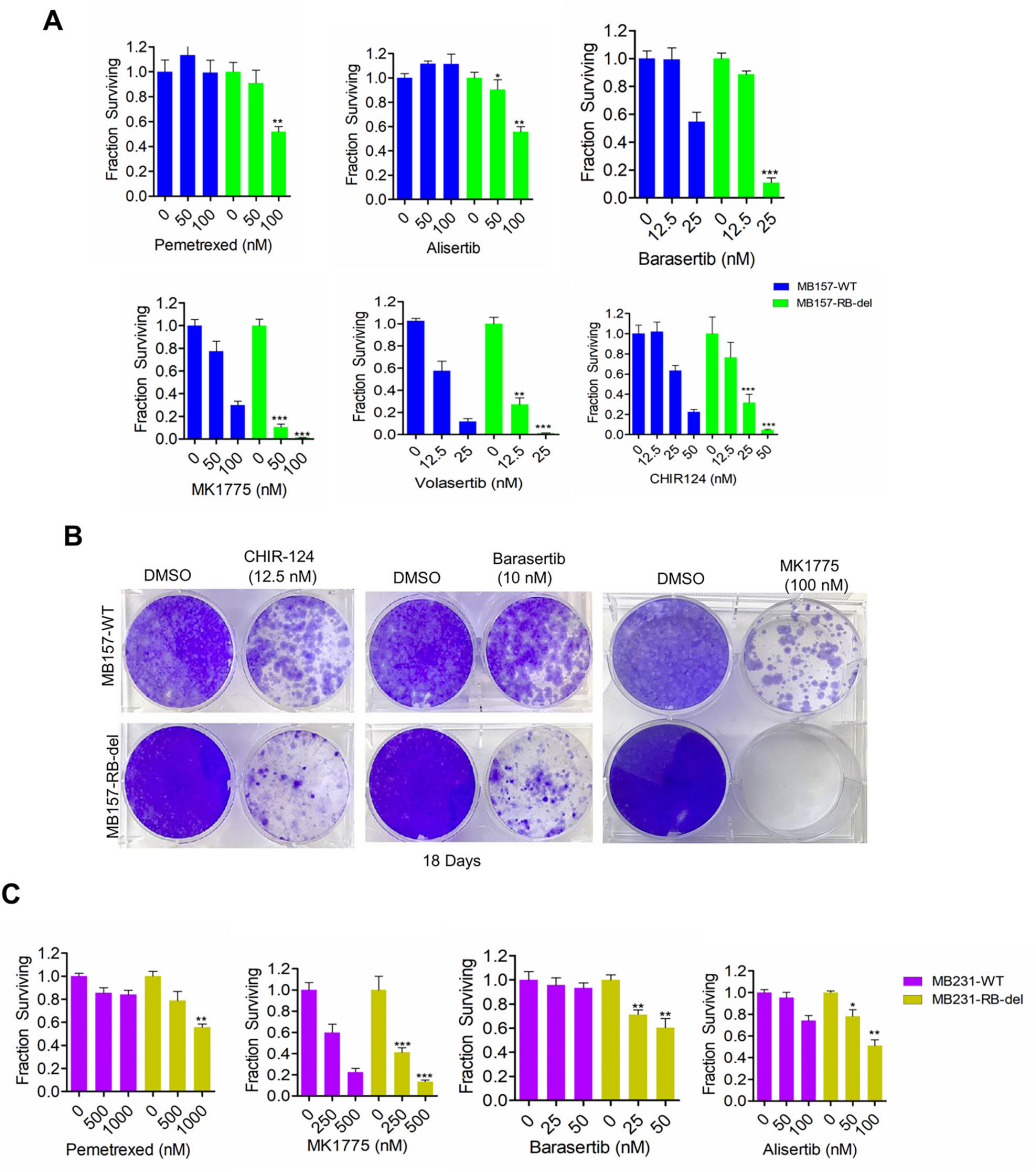
Single agent efficacy of the top drug hits from the drug screen. **A** Column graphs illustrating the viability of MB157-WT and RB-del cells as determined by the CellTiter Glo (CTG) assay following treatment with the indicated drugs at different concentrations for five days. Error bars represent mean and SD from triplicates. The experiments were done three independent times. (***p < 0.0001, **p < 0.001, *p < 0.05 as determined by student t-test). **B** Crystal violet staining on the resulting colonies formed after long-term treatment with CHIR-124 (12.5 nM), barasertib (10 nM) and MK1775 (100 nM) for 18 days in MB157-WT and RB-del cells. **C** Cell viability in MB231-WT and RB-del cells were determined by the CTG assay following five days of treatment with the indicated drugs at indicated concentrations. Error bars were determined based on mean and SD from triplicates. (***p < 0.0001, **p < 0.001, *p < 0.05 as determined by student t-test). Experiments were done three independent times.

To validate the observations from the drug screen, a dose response analysis was performed using a representative inhibitor from each drug class. Consistent with the drug screen, pemetrexed (DNA metabolism), alisertib (AURK), barasertib (AURK), MK1775 (WEE1), volasertib (PLK) and CHIR124 (CHK) exhibited enhanced cytotoxicity in RB-deficient MB157 cells with a significant shift in drug sensitivity (Fig. 2A). As a complementary approach, long-term colony formation assay confirmed that RB loss increases vulnerability to CHIR-124, barasertib and MK1775 (Fig. 2B). In MB231 cells, RB-loss modestly enhanced the efficacy of pemetrexed, MK1775, alisertib and barasertib at a higher concentration range as compared to MB157 cells (Fig. 2C). Overall, these data confirm that RB loss increases sensitivity to multiple cell cycle-targeting agents, while the degree of single agent efficacy varies depending on the cellular context.

### Combinatorial approach yields synthetic lethality

**Fig. 3:**
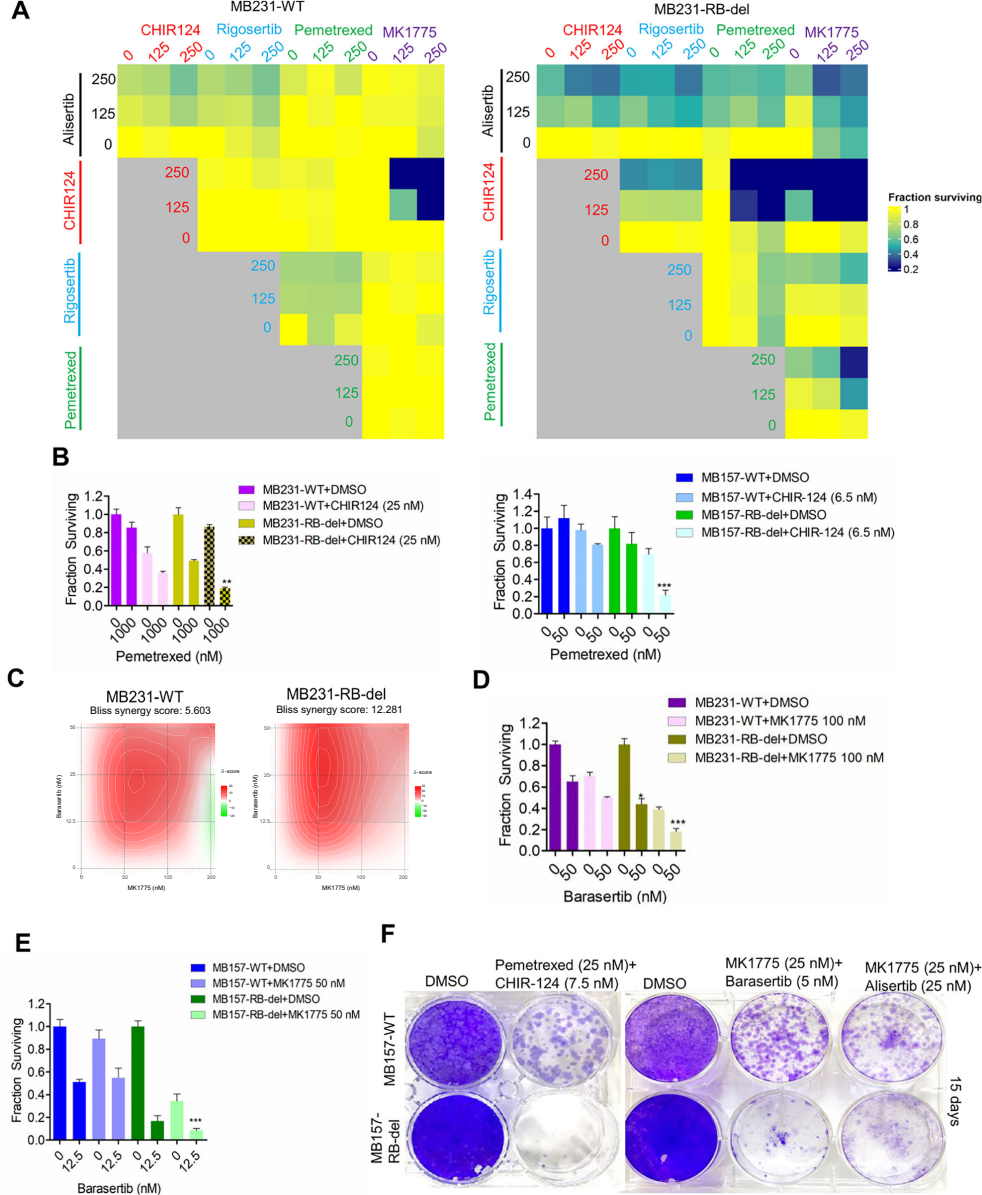
Combinaton treatments induce selective cell killing in RB-deleted models. **A** Heat maps depicting the synergistic interactions between the indicated pairs of drugs from MB231-WT and MB231-RB-del cells following five days of treatment. The fraction surviving was determined by the CellTiter Glo (CTG) assay. **B** Column graphs illustrate the viability of MB157-WT, MB157- RB-del, MB231-WT and MB231-RB-del cells following treatment with pemetrexed in combination with DMSO and CHIR-124. Error bars represent mean and SD from triplicates. Experiment was done at three independent times. ***p < 0.0001 and **p < 0.001 as determined by student t-test. **C** Bliss Synergy plots illustrating the % inhibition of cell viability following combination treatment with barasertib and MK1775 at the indicated doses for four days in MB231-WT and MB231-RB-del cells. **D** Effect of barasertib at the indicated concentrations on the viability of MB231-WT and MB231-RB-del cells in combination with MK1775 following exposure for 5 days. Error bars represent mean and SD from triplicates. (***p < 0.0001, **p < 0.001, *p < 0.05 as determined by student *t* test). **E** Column graphs indicating the fraction of survival cells following the treatment with barasertib in combination with MK1775 in MB157-WT and RB-del cells. Error bars represent mean and SD from triplicates. (***p < 0.0001 as determined by student *t* test). **F** Long-term effects of pemetrexed (25 nM) + CHIR124 (6.5 nM), MK1775 (25 nM) + barasertib (5 nM) and MK1775 (25 nM) + alisertib (50 nM) on the colony forming potential of MB157-WT and MB157-RB-del cells following 15 days of treatment.

Given the limited efficacy of single-agent treatments in inducing selective lethal effects in RB-del MB231 cells, combinatorial approaches were investigated for enhanced cell death, particularly in the context of RB-deletion. To define a potential combination treatment, the MB231-WT and RB-del cells were subjected to a combinatorial drug screen where the inhibitors from each drug class were tested in all possible pairwise combinations (Fig. 3A). Among different combinatorial pairs, concurrent administration of pemetrexed and CHIR124 synergistically reduced cell viability selectively in the MB231-RB-del cells (Fig. 3A). The combination of pemetrexed and CHIR124 yielded an enhanced cell death, which is significantly more potent in the RB-deficient MB231 and MB157 cells (Fig. 3B). Additionally, the concurrent inhibition of WEE1 and AURKA kinases using MK1775 and barasertib, respectively, yielded a synergistic interaction selectively in MB231-RB-del cells (Fig. 3C). This combination treatment further resulted in cell killing, which was significantly more potent in the RB-del MB157 and MB231 cells as compared to the RB-proficient wildtype cells (Fig. 3D, E). Long-term colony formation assays further validated that RB loss enhanced the efficacy of the indicated combination treatments and prominently reduced cell survival (Fig. 3F). This data demonstrates that co-targeting different cell cycle processes that are regulated by RB leads to synthetic lethality selectively in the RB-deficient setting.

### Cellular consequences of the combination treatments in TNBC models

**Fig. 4:**
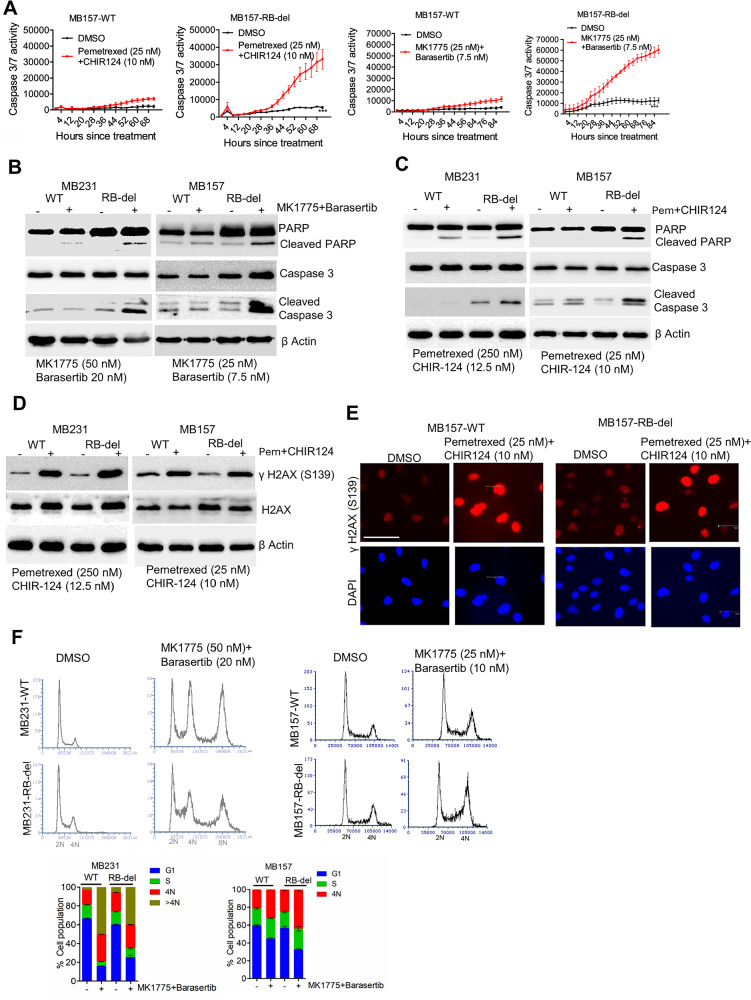
Mechanistic impact of different combination treatments. **A** Live cell imaging to monitor caspase-3/7 activity in real time in MB157-WT and MB157-RB-del cells following treatment with pemetrexed + CHIR124 and MK1775 + barasertib for 68 and 84 h, respectively. Error bars represent mean and SEM from triplicates. Experiment was done two independent times. (***p < 0.0001 as determined by two-way ANOVA). **B** Biochemical analysis on the indicated proteins from MB231-WT, MB231-RB-del, MB157-WT and MB157-RB-del following 48 h exposure with MK1775+Barasertib. **C** Effect of Pemetrexed+CHIR124 on the Cleaved PARP and cleaved caspase 3 from MB231-WT, MB231-RB-del, MB157-WT and MB157-RB-del cells following 48-hour incubation. **D** Immunoblot analysis to demonstrate the effect of Pemetrexed+CHIR124 on γH2AX in MB231-WT, MB231-RB-del, MB157-WT and MB157-RB-del cells. **E** Effect of pemetrexed+CHIR124 on the γH2AX foci formation in MB157-WT and MB157-RB-del cells following 48-hour exposure as determined by immunofluorescence. Scale bar represents 40 microns. **F** Cell cycle analysis in MB231-WT, MB231-RB-del, MB157-WT, and MB157-RB-del cells following treatment with MK1775 + barasertib for 48 h. The stack plots illustrate the % cell population at each phase of the cell cycle.

Due to the observed impact on cell survival in RB-deficient cells following the different combination treatments, the apoptotic pathway was further evaluated. Live cell imaging was used to monitor caspase 3/7 activity in real time in both MB157-WT and RB-del cells. While caspase 3/7 activity remained unchanged in MB157-WT cells following the treatment with MK1775+barasertib and pemetrexed+CHIR-124, a significant increase was observed in the RB-deficient cells (Fig. 4A). Biochemical analysis further confirmed that the combination treatments, MK1775+barasertib and pemetrexed+CHIR124 selectively activated apoptosis in the RB-deficient MB231 and MB157 cells as evidenced by enhanced cleavage of PARP and caspase-3, while the impact on these proteins were modest in the wild-type cells (Fig. 4B, C).

We then examined whether cell death in the RB-deficient cells following the combination treatments were a consequence of differential impact on cell cycle processes. Since pemetrexed inhibits DNA synthesis and CHIR124 targets CHK1, which is involved in DNA repair, the effect of this combination treatment on the DNA damage response in both RB-proficient and deficient settings was examined [31–33]. The combination treatment resulted in DNA damage as evident by the induction of γH2AX in both the MB231 and MB157 cells in an RB-independent manner (Fig. 4D). Immunofluorescence assay further confirmed the γH2AX foci formation in both MB157-WT and RB-del cells (Fig. 4E). Similarly, the combination treatment involving MK1775 and barasertib resulted in a significant accumulation of cells with 4 N and 8 N DNA content in both the wild-type and RB-deficient MB231 and MB157 cells (Fig. 4F). This indicates a cell cycle block, which is post DNA replication, consistent with the cellular functions of their target proteins, WEE1 and Aurora Kinases, respectively [34, 35]. These data illustrate that while the combination treatments induce comparable cell cycle arrest and DNA damage in both RB-proficient and deficient cells, RB loss selectively enhances the cellular susceptibility to apoptosis.

### Defining the apoptotic pathway in RB-deficient cells for therapeutic intervention

**Fig. 5:**
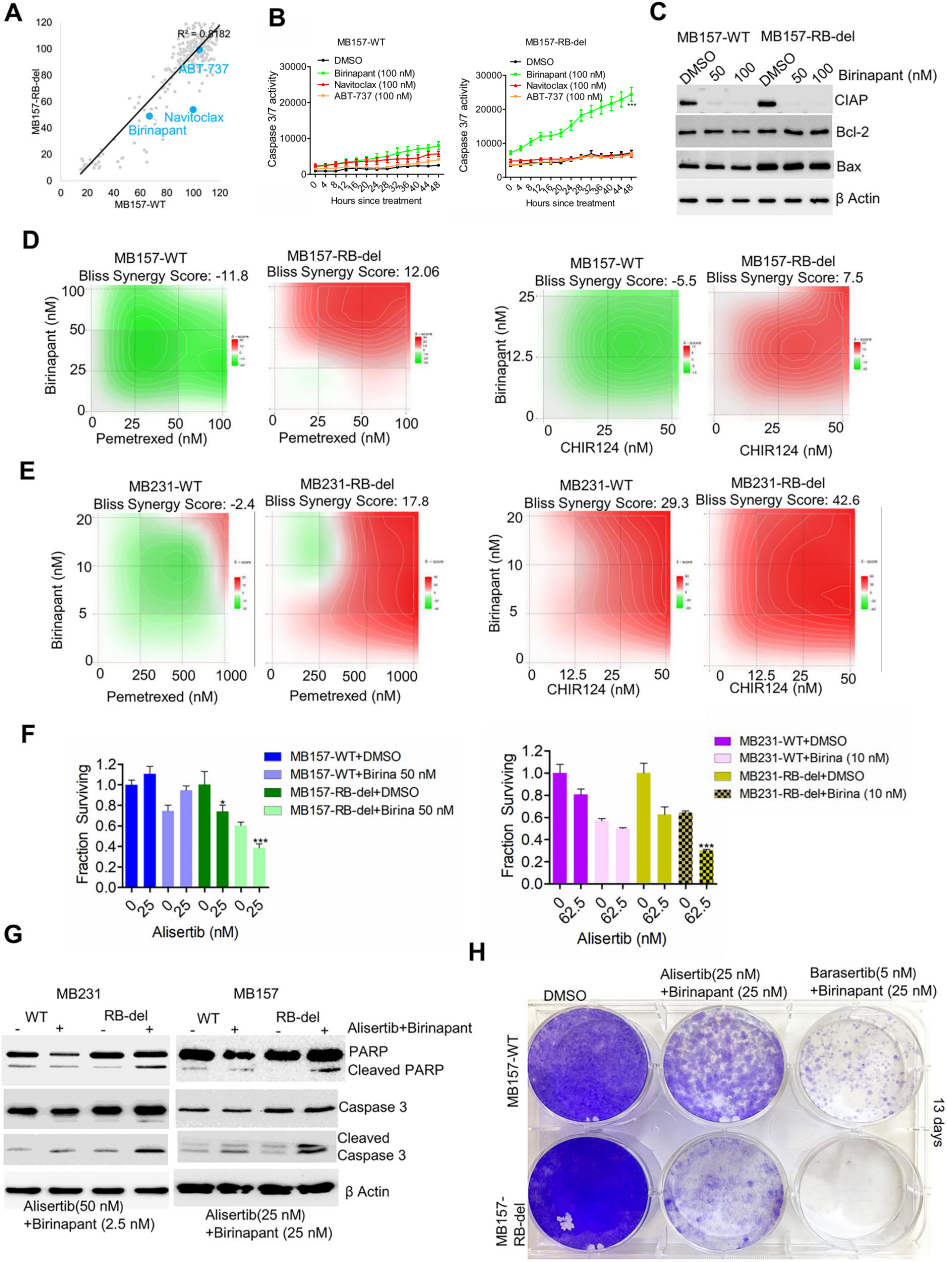
Therapeutically targeting the apoptosis pathway induces lethal effect in RB-deleted models. **A** Correlation plot from the drug screening to depict the differential effects of birinapant, navitoclax and ABT-737 between MB157-WT and MB157-RB-del cells. **B** Live cell imaging approach to monitor the caspase-3/7 activity in MB157-WT and MB157-RB-del cells following treatment with different apoptotic drugs such as birinapant, navitoclax and ABT-737 at 100 nM. The error bars represent mean and SEM from triplicates. The experiment was done two independent times (***p < 0.0001 as determined by 2-way ANOVA). **C** Western blotting on MB157-WT and RB-del cells to examine the expression of CIAP, Bcl2 and Bax following treatment with birinapant at the indicated concentrations for 48 h. **D** Combination treatment involving birinapant + pemetrexed and birinapant + CHIR-124 at different concentrations in MB157-WT and RB-del cells. The synergistic interaction was determined by Synergy finder where the Bliss Synergy score was calculated. **E** Synergistic effect on cell death following the treatment with birinapant + pemetrexed and birinapant + CHIR-124 in MB231-WT and RB-del cells. The synergistic interaction was determined by Synergy finder where the Bliss Synergy score was calculated. **F** Column graphs illustrating the effect of alisertib on cell viability in MB157-WT, MB157-RB-del, MB231-WT, and MB231-RB-del cells in combination with DMSO and birinapant following five days of treatment. Error bars were determined based on mean and SD from triplicates. The experiment was done three independent times. (***p < 0.0001, *p < 0.05 as determined by student *t* test). **G** Biochemical analysis to determine the effect of alisertib + birinapant on PARP cleavage and caspase 3 cleavage in MB231-WT, MB231-RB-del, MB157-WT and MB157-RB-del cells. **H** Long-term colony formation assay in MB157-WT and RB-del cells following treatment with alisertib + birinapant and barasertib+birinapant for 13 days.

To investigate the pathways mediating apoptosis, we examined our drug screen for agents targeting distinct apoptotic mechanisms. Among the drugs that induce apoptosis, we identified birinapant yielded a selective effect in the MB157-RB-del cells as compared to its wild-type counterpart (Fig. 5A). Birinapant is a SMAC mimetic, which promotes degradation of the anti-apoptotic protein CIAP to activate TNFα signaling-mediated apoptosis [36–38]. To validate this finding we compared the efficacy of birinapant with different pharmacological agents, navitoclax and ABT-737 that induce mitochondrial apoptosis via Bcl-2 inhibition [39–41]. Among the different drugs tested, the Bcl-2 inhibitors possessed modest effects on apoptosis induction in both MB157-WT and RB-del cells as monitored by real time caspase 3/7 activity (Fig. 5B). Interestingly, birinapant, which had only a modest effect on the MB157-WT cells, significantly enhanced caspase 3 activity in the MB157-RB-del cells (Fig. 5B). Biochemical analysis revealed that birinapant did not alter the levels of Bcl-2 and Bax that regulate the intrinsic apoptotic pathway (Fig. 5C). However, birinapant degraded its intracellular target, CIAP in both MB157-WT and MB157-RB-del cells, suggesting its therapeutic impact on the extrinsic TNF signaling (Fig. 5C). Overall, our data indicates that, while the on-target efficacy of birinapant is RB independent, its ability to trigger apoptosis occurred selectively in the RB-del MB157 cells.

We then explored whether the cellular response to birinapant could be exploited to yield synthetic lethality in RB-deficient settings. Since pemetrexed and CHIR124 were more potent in RB-deficient cells, their impact on cell survival in combination with birinapant was evaluated. In both MB157 and MB231 cells, a synergistic interaction was observed when birinapant was combined with pemetrexed and CHIR124, and this synergy was more potent in the RB-deficient models, suggesting that RB loss induces a synthetic lethal effect (Fig. 5D, E). Similarly, birinapant in combination with the AURK inhibitor alisertib was significantly more potent and selectively lethal in the RB deficient MB157 and MB231 cells as compared to its wildtype counterparts (Fig. 5F). Biochemical analysis in MB231 and MB157 cells revealed that the combination of alisertib and birinapant selectively activated apoptosis in the RB-deficient cells as evident by the induction of cleaved-PARP and cleaved caspase 3 (Fig. 5G). Finally, colony formation assay demonstrated that birinapant in combination with AURK inhibitors, alisertib and barasertib inhibited cell proliferation more potently in the MB157-RB-del cells as compared to their wild-type counterpart (Fig. 5H). Therefore, these findings demonstrate that pharmacologically targeting the apoptotic pathway, particularly via TNFα signaling, elicits a promising therapeutic strategy for RB-deficient tumors.

### RB loss drives synthetic lethality in vivo

**Fig. 6:**
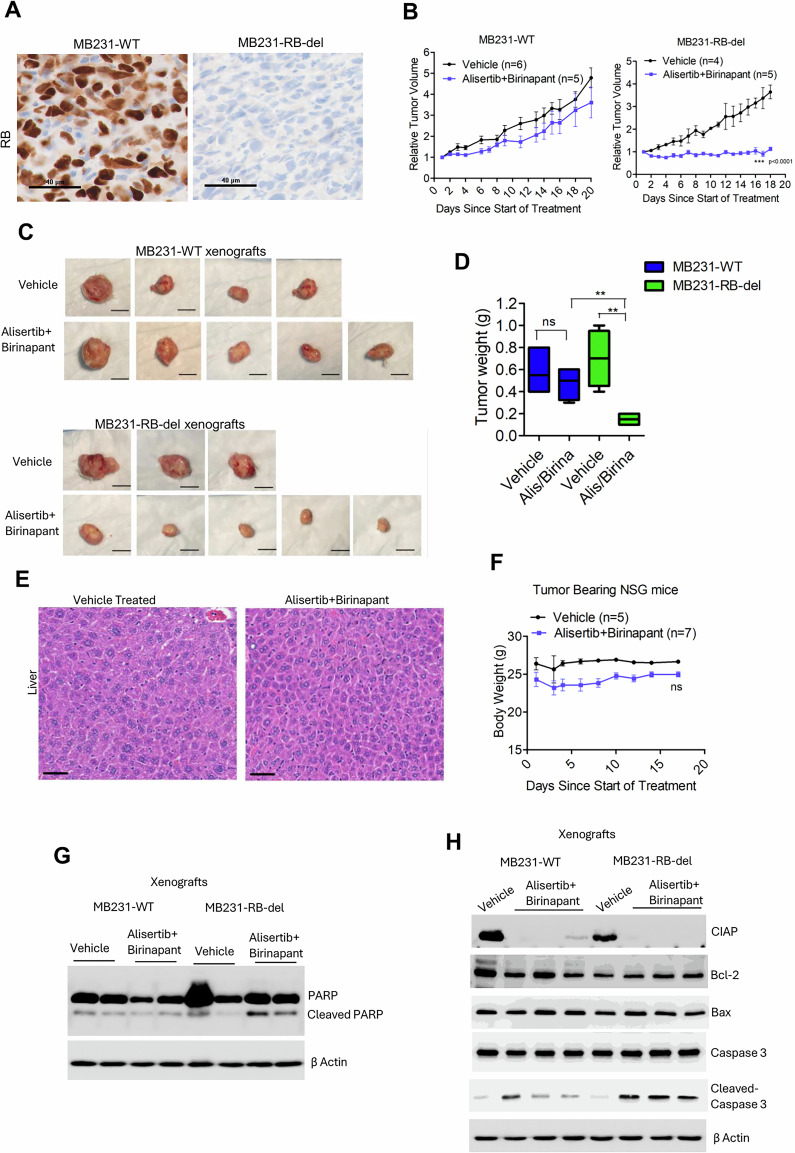
RB loss enhances in vivo anti-tumor response to alisertib+birinapant. **A** Representative immunohistochemical images on the tissues derived from MB231-WT and RB-del xenografts to validate RB expression. The scale bar represents 40 µm. **B** Differential effect of alisertib (10 mg/kg) in combination with birinapant (15 mg/kg) on tumor growth derived from MB231-WT and MB231-RB-del xenografts. Error bars represent mean and SEM. (***p < 0.0001 as determined by 2-way ANOVA). **C** Representative tumor images from MB231-WT and MB231-RB-del xenografts that were treated with vehicle or alisertib + birinapant. **D**. Tumor weights from MB231-WT and MB231-RB-del xenografts that were treated with vehicle or alisertib + birinapant. Error bars represent mean and SEM. (**p < 0.001 as determined by unpaired student *t* test). **E** H&E staining on the liver from tumor-bearing NSG mice treated with vehicle and alisertib+birinapant. **F** Effect of alisertib+birinapant on the mice body weights during the course of treatment. **G** Biochemical analysis to determine the in vivo effect of alisertib + birinapant in inducing cleaved PARP on the MB231-WT and MB231-RB-del xenografts. **H** Immunoblotting from the tumor tissues excised from mice bearing xenografts derived from MB231-WT and MB231-RB-del cells to determine the expression of CIAP, Bcl-2, Bax and cleaved caspase 3 following treatment with vehicle or alisertib + birinapant.

To interrogate whether cell death driven by RB loss in cell culture assays could be translated in vivo, we established xenografts derived from the isogenic cell lines, MB231-WT and RB-del cells. Immunohistochemical staining for RB from the tumor tissues confirmed the retention of the RB-deficient phenotype in vivo (Fig. 6A). The mice were treated with a low-dose combination of alisertib and birinapant to limit the single-agent anti-tumor efficacy as demonstrated in our previous study [17]. In the RB-proficient MB231 xenografts, the combination treatment did not elicit a prominent impact on tumor growth (Fig. 6B). However, RB loss significantly enhanced tumor susceptibility to the combination treatment, leading to inhibition of tumor growth and reduced tumor size (Fig. 6B). The combination treatment yielded significant disease control in the MB231-RB-del xenografts as compared to the MB231-WT xenografts (Fig. 6C, D). To examine the potential off-target toxicity of alisertib + birinapant, histological analyses of the liver was performed, which revealed no prominent change in liver morphology (Fig. 6E). Moreover, no significant differences in body weight were observed between vehicle- and alisertib + birinapant–treated groups, indicating that the combination treatment is well tolerable (Fig. 6F).

To assess the in vivo outcomes of alisertib+birinapant biochemical analysis from the tumor tissues were performed. The induction of cleaved PARP following the combination treatment was observed selectively in the MB231-RB-del tumors as compared to their wild-type counterparts, indicating apoptotic cell death in the RB-deficient setting (Fig. 6G). Further mechanistic investigation revealed that the in vivo combination treatment did not alter the intrinsic apoptotic pathway based on the intact levels of Bcl-2 and Bax, which is consistent with the cell culture data (Fig. 6H). The reduced expression of CIAP in both MB231-WT and RB-del xenografts, confirms the in vivo on-target efficacy of birinapant and renders the RB-deficient tumors to undergo cell-death, which is further confirmed by induction of cleaved caspase-3 (Fig. 6H). Together, these findings indicate that RB loss enhances susceptibility to apoptotic cell death in vivo leading to tumor growth suppression.

## Discussion

RB loss is observed in approximately 30% of TNBC cases and represents a key mechanism of resistance to targeted therapies that converge on the cell cycle machinery. The loss of RB function uncouples the cell cycle from CDK4/6 kinase regulation, rendering the cells unresponsive to pharmacological agents that target these kinases or their upstream mitogenic pathways. As a result, therapeutic strategies that rely on RB activation often limit clinical benefit in RB-deficient TNBC, leading to therapy resistance. Given this challenge, we aimed to uncover therapeutic vulnerabilities that emerge specifically in the context of RB loss and could be leveraged to induce selective lethality in RB-deficient TNBC.

An unbiased pharmacological drug screening was employed to identify therapeutic strategies that selectively impact RB-deficient tumor models. The robustness of the screen is underscored by the consistent identification of multiple drugs against the same targets, including Aurora kinase, Polo-like Kinase and CHK1, emphasizing the functional relevance of their associated pathways in the RB-deficient context. These findings demonstrate distinct vulnerabilities in the RB-deficient setting, which could be therapeutically exploited by targeting DNA replication, repair mechanisms and mitotic checkpoints.

The cellular targets identified from the drug screen represent essential regulators of cell cycle progression and survival, raising concerns about potential adverse effects due to the inhibition of these pathways in non-cancerous cells. To overcome this, a combinatorial treatment strategy was employed that allowed for dose reduction of each drug while achieving a synergistic therapeutic effect. This approach was synthetically lethal in RB-deficient cells, where co-targeting these critical pathways led to robust apoptotic cell death. Cell-based analysis revealed that inhibition of DNA repair or mitotic progression induced cellular stress by disrupting the cell cycle, a response observed irrespective of RB status. However, the activation of apoptosis in response to this stress was significantly faster in RB-deficient cells. This differential apoptotic response was quantified in real time using a fluorescence-based live-cell imaging approach that tracks caspase 3 activity, allowing precise measurement of apoptotic kinetics. These findings uncover a non-canonical role for RB, where its loss primes cells for apoptosis in response to stress signals.

Cellular apoptosis is regulated through two major pathways: (i) the intrinsic (mitochondrial) pathway, where pro-apoptotic Bcl-2 family proteins disrupt mitochondrial membrane potential, leading to cytochrome c release and activation of caspase 3; and (ii) the extrinsic pathway, where death ligands such as TNFα bind to cell surface receptors like TNFR1 to activate downstream signaling that initiates apoptosis [42–44]. Using a pharmacological approach, we found that RB loss preferentially sensitizes tumor cells to the extrinsic apoptotic pathway. The cellular inhibitor of apoptosis proteins (cIAPs), ubiquitylate RIPK1 and inhibits its function and serve as negative regulators of TNF-mediated apoptosis [24]. Birinapant, a SMAC mimetic, targets and depletes cIAPs, stabilizing RIPK1 and promoting apoptosis in response to TNF signaling [38]. Previous studies have shown that RB degradation is a prerequisite for TNF-induced apoptosis, suggesting a mechanistic link between RB status and extrinsic pathway activation [45, 46]. Building on this, we developed a synthetic lethal approach in RB-deficient models where the apoptotic response to cellular stress following the inhibition of cell cycle checkpoints could be further potentiated using birinapant. The rationale for this is supported from our previous study in ER+ breast cancer models where the treatment with AURK and WEE1 kinase inhibitors resulted in the upregulation of TNFα signaling [17]. Consistently, the combination approach involving birinapant induced a selective anti-tumor response in RB-deficient models in vivo. In conclusion, our study reveals that RB loss creates a unique vulnerability to extrinsic apoptotic signaling and demonstrates how this vulnerability can be therapeutically exploited using rational drug combinations to selectively eliminate RB-deficient tumors.

## Methods

### Cell culture and reagents

The triple-negative breast cancer (TNBC) cell lines MB231 and MB157 were cultured in Dulbecco’s Modified Eagle Medium (DMEM) supplemented with 10% fetal bovine serum (FBS) and maintained at 37 °C in a humidified incubator with 5% CO₂. The cell lines were authenticated by STR profiling and confirmed to be Mycoplasma free based on PCR and DAPI staining. A drug library consisting of 305 compounds was purchased from SelleckChem, each provided at a stock concentration of 10 mM as described in our previous study [22]. Additional compounds, including alisertib and birinapant, were obtained from MedChem Express and similarly dissolved in DMSO to a final stock concentration of 10 mM.

### Generation of isogenic RB-deleted cell lines

MB157 and MB231 cells were infected with lenti-viral particles that contain the vector, pL-CRIPSR-EFS-sgRB-tRFP, which carries the guide RNA sequence, 5’- GGTTCTTTGAGCAACATGGG that targets the human *RB1* gene. The deletion of *RB1* was validated using an immunofluorescence assay that assessed the expression of the RB protein.

### Immunofluorescence

Cells were seeded on coverslips and incubated for 48 h, then fixed with 4% formaldehyde at room temperature for 15 min. Following fixation, cells were permeabilized using a buffer containing 1% bovine serum albumin (BSA) and 0.5% Triton X-100. Primary antibodies, including anti-RB (RB4H1) (Cell Signaling Technology; 9309 L) and anti- γH2AX (S139) (Cell Signaling Technology; SC-9966), were diluted 1:50 in antibody diluent (5% BSA and 0.5% NP-40 in PBS), and cells were incubated with this mixture for 1 h at room temperature. The secondary antibody used was a fluorophore-conjugated donkey anti-rabbit antibody and used in 1:100 dilution. The nucleus was stained using DAPI. Fluorescent signals were imaged using an EVOS FL Auto 5000 imaging system.

### Western blotting

Whole cell extract was prepared using RIPA lysis buffer (10 mM Tris HCl, pH 8.0, 1 mM EDTA, 150 mM NaCl, 1% Triton-X-100, 0.1% sodium deoxycholate, 0.1% SDS) supplemented with Halt Protease inhibitor cocktail and 1 mM PMSF as described in our previous studies [30, 47]. The extracted proteins were resolved on a 12% SDS-PAGE gel and transferred to a nitrocellulose membrane. The membranes were incubated at 4 °C overnight with primary antibodies, anti-pRB (S807/811) (Cell Signaling Technology; 8516S), RB-4H1 (Cell Signaling Technology; 9309), anti-cleaved caspase 3(Cell Signaling Technology; 9661), anti-caspase 3 (Cell Signaling Technology; 9662), anti-Bax (Cell Signaling Technology; 2772), anti-Bcl-2 (Thermo scientific; 13-8800), anti-PARP (Cell Signaling Technology; 9542S), anti-CIAP (Cell Signaling Technology), and anti-β-Actin (Cat #MAB8929, R&D Systems Minneapolis, MN) diluted 1:1500 in the antibody diluent solution (5% BSA in 25 mM Tris HCl, pH 7.5, 150 mM NaCl, 0.1% Tween 20). The membranes were further incubated with HRP-conjugated secondary antibodies that were purchased from Santa Cruz Biotechnology at room temperature for 1 h. The luminescence signal was visualized using an Enhanced ECL substrate (Thermo Fisher Scientific) on the Bio-Rad imager.

### Cell cycle analysis

Cells were seeded in 6-well tissue culture (TC) dish and exposed to different therapeutic agents for 48 h. Cells were harvested using trypsin and the cell pellets were fixed in freshly prepared ice-cold 70% ethanol for 30 min at 4 °C. Following fixation, the cells were washed with 1X phosphate-buffered saline (PBS) and treated with RNaseA (200 µg/mL) and a nucleic acid binding dye Propidium Iodide (PI) (1.6 mg/mL). The cell cycle analysis was performed on the BD LSRFORTESSA flow cytometer. Histograms illustrating the distinct cell cycle phases were generated using the FCS express software as described in our previous study [30].

### Caspase 3/7 activity and cell viability assay

Live cell imaging was used to monitor caspase-3 activity in real time. Cells were seeded into a 96-well TC dish at a density of 1500 cells/well. After 24 h, the medium was replaced with fresh medium containing the CellEvent Caspase 3/7 Red Detection Reagent (Thermo Fisher Scientific Cat # C10430) at the dilution suggested in the protocol along with the test compounds and DMSO. Apoptosis was determined based on the red fluorescence intensity using the IncuCyte live-cell imaging system. The rate of increase in apoptosis was analyzed in GraphPad Prism. As an endpoint assay, cell viability was measured using CellTiter Glo (Promega) according to the manufacturer’s protocol.

### Drug screening

The cells were seeded in a 384-well TC dish using an automatic dispenser. Following 24-h incubation, the cells were exposed to a drug library comprised of 305 compounds as described in our previous studies [17, 30]. The cells were treated with the drug library for 96 h and the cell viability was determined using the CTG assay. The pair-wise combination screening was performed by seeding the cells in a 384-well plate. After 24 h, the different drug pairs were dispended using a drug printer at different concentrations and incubated for another 96 h. The cell viability was determined by the CTG assay and the synergistic interaction between the drug pairs were determined using the online Synergy Finder Platform to calculate the Bliss Synergy score.

### Clonogenic survival assay

To examine the long-term efficacy of the drugs, cells were seeded in a six-well TC dish at a density of 5000 cells/well. Following 24-h incubation, cells were exposed to different drugs either as single agents or in combination. The cells were allowed to form colonies for up to 15–21 days and the drug containing medium was replaced every five days. The resulting colonies were stained with Crystal violet dye (2% Crystal violet and 5% methanol) according to the standard procedure.

### In vivo xenografts

NOD scid gamma (NSG) mice were maintained at Roswell Park Comprehensive Cancer Center animal care facilities. All animal care, drug treatments, and method of euthanasia were approved by the Roswell Park Comprehensive Cancer Center Institutional Animal Care and Use Committee (IACUC) in accordance with the NIH guidelines for the care and use of laboratory animals. Eight to ten week old female NSG mice were subcutaneously injected with the early passage MB231-WT and MB231-RB-del cells (1 × 10^7^ cells/mouse). Following ten days of injection, mice were monitored for palpable tumor formation. Once the tumor volume reached 150–200 mm^3^, mice were randomized to different treatment cohorts as follows in a non-blinded manner. Vehicle (n = 10) (30% PEG300 + 5%Tween 80 + ddH_2_O) was administered orally. For the combination group (n = 10), Alisertib (10 mg/kg) (30% PEG300 + 5%Tween 80 + ddH_2_O) was administered orally for six days a week for three weeks. Birinapant (15 mg/kg) (40% PEG300 + 5% Tween 80 + 45% saline + 10% DMSO) was administered via IP injection for three days/week up to three weeks as described in our previous study [17]. Tumor size was measured every day using digital calipers and the volume was calculated using the formula: (greatest diameter*(shortest diameter^2^))/2. Mice were sacrificed when the tumor size reached 2000 mm^3^. Tumor tissues were excised and subjected to protein extraction using RIPA lysis buffer (10 mM Tris HCl, pH 8.0, 1 mM EDTA, 150 mM NaCl, 1% Triton-X-100, 0.1% sodium deoxycholate, 0.1% SDS) supplemented with Halt Protease inhibitor cocktail and 1 mM PMSF for Western blot analysis. Mice that became sick or found dead during the course of treatment were excluded from the analysis.

### Immunohistochemistry

The excised tumor tissues were fixed in 10% formalin followed by processing and paraffin embedding. Immunohistochemical staining was performed using the anti-RB4H1 antibody (Cat # 9309) (Cell signaling Technologies). Slides were scanned using the Akoya PhenoImager (Akoya Biosciences).

### Statistical analysis

All the plate-based assays including the CTG and caspase-3/7 activity were done in triplicates, and the experiments were done three independent times. In all the assays the error bars were determined using mean and SD or SEM, with the variance being similar between the groups. Statistical analyses were performed using unpaired student t-test and two-way ANOVA.

## Supplementary information

Supplementary Information

## Data Availability

The original and uncropped western blots are provided as supplemental material. Other data will be available from the corresponding author on reasonable request.

## References

[1] Borri F, Granaglia A. Pathology of triple negative breast cancer. Semin Cancer Biol. 2021;72:136–45. 32544511 10.1016/j.semcancer.2020.06.005

[2] Foulkes WD, Smith IE, Reis-Filho JS. Triple-negative breast cancer. N. Engl J Med. 2010;363:1938–48. 21067385 10.1056/NEJMra1001389

[3] Wang L, Zhai Q, Lu Q, Lee K, Zheng Q, Hong R, et al. Clinical genomic profiling to identify actionable alterations for very early relapsed triple-negative breast cancer patients in the Chinese population. Ann Med. 2021;53:1358–69. 34396843 10.1080/07853890.2021.1966086 PMC8381897

[4] Cardoso F, Kyriakides S, Ohno S, Penault-Llorca F, Poortmans P, Rubio IT, et al. Early breast cancer: ESMO Clinical Practice Guidelines for diagnosis, treatment and follow-up. Dagger Ann. Oncol. 2019;30:1194–220. 10.1093/annonc/mdz173 31161190

[5] El Gazzar WB, Albakri KA, Hasan H, Badr AM, Farag AA, Saleh OM. Poly(ADP-ribose) polymerase inhibitors in the treatment landscape of triple-negative breast cancer (TNBC). J Oncol Pharm Pract. 2023;29:1467–79. 37559370 10.1177/10781552231188903

[6] Fong PC, Yap TA, Boss DS, Carden CP, Mergui-Roelvink M, Gourley C, et al. Poly(ADP)-ribose polymerase inhibition: frequent durable responses in BRCA carrier ovarian cancer correlating with platinum-free interval. J Clin Oncol. 2010;28:2512–9. 20406929 10.1200/JCO.2009.26.9589

[7] Pauwels EKJ, Bourguignon MH. PARP inhibition and beyond in BRCA-associated breast cancer in women: a state-of-the-art summary of preclinical research on risk reduction and clinical benefits. Med Princ Pract. 2022;31:303–12. 35636395 10.1159/000525281 PMC9485988

[8] Hoxhaj G, Manning BD. The PI3K-AKT network at the interface of oncogenic signalling and cancer metabolism. Nat Rev Cancer. 2020;20:74–88. 31686003 10.1038/s41568-019-0216-7 PMC7314312

[9] Sharma P, Abramson VG, O’Dea A, Nye L, Mayer I, Pathak HB, et al. Clinical and biomarker results from phase I/II study of PI3K inhibitor alpelisib plus Nab-paclitaxel in HER2-negative metastatic breast cancer. Clin Cancer Res. 2021;27:3896–904. 33602685 10.1158/1078-0432.CCR-20-4879 PMC8282704

[10] Wang R, Xu K, Gao F, Huang J, Guan X. Clinical considerations of CDK4/6 inhibitors in triple-negative breast cancer. Biochim Biophys Acta Rev Cancer. 2021;1876:188590. 34271137 10.1016/j.bbcan.2021.188590

[11] Cottu P, D’Hondt V, Dureau S, Lerebours F, Desmoulins I, Heudel PE, et al. Letrozole and palbociclib versus chemotherapy as neoadjuvant therapy of high-risk luminal breast cancer. Ann. Oncol. 2018;29:2334–40. 30307466 10.1093/annonc/mdy448

[12] Turner NC, Ro J, Andre F, Loi S, Verma S, Iwata H, et al. Palbociclib in hormone-receptor-positive advanced breast cancer. N Engl J Med. 2015;373:209–19. 26030518 10.1056/NEJMoa1505270

[13] Turner NC, Slamon DJ, Ro J, Bondarenko I, Im SA, Masuda N, et al. Overall survival with palbociclib and fulvestrant in advanced breast cancer. N Engl J Med. 2018;379:1926–36. 30345905 10.1056/NEJMoa1810527

[14] Hortobagyi GN, Stemmer SM, Burris HA, Yap YS, Sonke GS, Hart L, et al. Overall survival with ribociclib plus letrozole in advanced breast cancer. N Engl J Med. 2022;386:942–50. 35263519 10.1056/NEJMoa2114663

[15] Zagami P, Carey LA. Triple negative breast cancer: Pitfalls and progress. NPJ Breast Cancer. 2022;8:95. 35987766 10.1038/s41523-022-00468-0 PMC9392735

[16] Chica-Parrado MR, Kim GM, Uemoto Y, Napolitano F, Lin CC, Ye D, et al. Combined inhibition of CDK4/6 and AKT is highly effective against the luminal androgen receptor (LAR) subtype of triple negative breast cancer. Cancer Lett. 2024;604:217219. 39244005 10.1016/j.canlet.2024.217219 PMC11837982

[17] Kumarasamy V, Nambiar R, Wang J, Rosenheck H, Witkiewicz AK, Knudsen ES. RB loss determines selective resistance and novel vulnerabilities in ER-positive breast cancer models. Oncogene. 2022;41:3524–38. 35676324 10.1038/s41388-022-02362-2 PMC10680093

[18] Rubin SM, Sage J, Skotheim JM. Integrating old and new paradigms of G1/S control. Mol Cell. 2020;80:183–92. 32946743 10.1016/j.molcel.2020.08.020 PMC7582788

[19] Sherr CJ. Cancer cell cycles. Science. 1996;274:1672–7. 8939849 10.1126/science.274.5293.1672

[20] Zhang HS, Postigo AA, Dean DC. Active transcriptional repression by the Rb-E2F complex mediates G1 arrest triggered by p16INK4a, TGFbeta, and contact inhibition. Cell. 1999;97:53–61. 10199402 10.1016/s0092-8674(00)80714-x

[21] Knudsen ES, Nambiar R, Rosario SR, Smiraglia DJ, Goodrich DW, Witkiewicz AK. Pan-cancer molecular analysis of the RB tumor suppressor pathway. Commun Biol. 2020;3:158. 32242058 10.1038/s42003-020-0873-9 PMC7118159

[22] Kumarasamy V, Vail P, Nambiar R, Witkiewicz AK, Knudsen ES. Functional determinants of cell cycle plasticity and sensitivity to CDK4/6 inhibition. Cancer Res. 2021;81:1347–60. 33323381 10.1158/0008-5472.CAN-20-2275 PMC8026500

[23] Witkiewicz AK, Chung S, Brough R, Vail P, Franco J, Lord CJ, et al. Targeting the vulnerability of RB tumor suppressor loss in triple-negative breast cancer. Cell Rep. 2018;22:1185–99. 29386107 10.1016/j.celrep.2018.01.022 PMC5967622

[24] Witkiewicz AK, Kumarasamy V, Sanidas I, Knudsen ES. Cancer cell cycle dystopia: heterogeneity, plasticity, and therapy. Trends Cancer. 2022;8:711–25. 35599231 10.1016/j.trecan.2022.04.006 PMC9388619

[25] Li Z, Razavi P, Li Q, Toy W, Liu B, Ping C, et al. Loss of the FAT1 tumor suppressor promotes resistance to cdk4/6 inhibitors via the hippo pathway. Cancer Cell. 2018;34:893–905.e898. 30537512 10.1016/j.ccell.2018.11.006 PMC6294301

[26] Asghar US, Kanani R, Roylance R, Mittnacht S. Systematic review of molecular biomarkers predictive of resistance to CDK4/6 inhibition in metastatic breast cancer. JCO Precis Oncol. 2022;6:e2100002. 35005994 10.1200/PO.21.00002 PMC8769124

[27] Brough R, Gulati A, Haider S, Kumar R, Campbell J, Knudsen E, et al. Identification of highly penetrant Rb-related synthetic lethal interactions in triple negative breast cancer. Oncogene. 2018;37:5701–18. 29915391 10.1038/s41388-018-0368-z PMC6202330

[28] Dick FA, Goodrich DW, Sage J, Dyson NJ. Non-canonical functions of the RB protein in cancer. Nat. Rev. Cancer. 2018;18:442–51. 29692417 10.1038/s41568-018-0008-5 PMC6693677

[29] Kim S, Leong A, Kim M, Yang HW. CDK4/6 initiates Rb inactivation and CDK2 activity coordinates cell-cycle commitment and G1/S transition. Sci Rep. 2022;12:16810. 36207346 10.1038/s41598-022-20769-5 PMC9546874

[30] Kumarasamy V, Wang J, Roti M, Wan Y, Dommer AP, Rosenheck H, et al. Discrete vulnerability to pharmacological CDK2 inhibition is governed by heterogeneity of the cancer cell cycle. Nat Commun. 2025;16:1476. 39924553 10.1038/s41467-025-56674-4 PMC11808123

[31] Adjei AA. Pemetrexed (Alimta): a novel multitargeted antifolate agent. Expert Rev Anticancer Ther. 2003;3:145–56. 12722874 10.1586/14737140.3.2.145

[32] Tse AN, Rendahl KG, Sheikh T, Cheema H, Aardalen K, Embry M, et al. CHIR-124, a novel potent inhibitor of Chk1, potentiates the cytotoxicity of topoisomerase I poisons in vitro and in vivo. Clin Cancer Res. 2007;13:591–602. 17255282 10.1158/1078-0432.CCR-06-1424

[33] Neizer-Ashun F, Bhattacharya R. Reality CHEK: understanding the biology and clinical potential of CHK1. Cancer Lett. 2021;497:202–11. 32991949 10.1016/j.canlet.2020.09.016

[34] Marumoto T, Hirota T, Morisaki T, Kunitoku N, Zhang D, Ichikawa Y, et al. Roles of aurora-A kinase in mitotic entry and G2 checkpoint in mammalian cells. Genes Cells. 2002;7:1173–82. 12390251 10.1046/j.1365-2443.2002.00592.x

[35] Sun L, Moore E, Berman R, Clavijo PE, Saleh A, Chen Z, et al. WEE1 kinase inhibition reverses G2/M cell cycle checkpoint activation to sensitize cancer cells to immunotherapy. Oncoimmunology. 2018;7:e1488359. 30288354 10.1080/2162402X.2018.1488359 PMC6169577

[36] Wilson WH, O’Connor OA, Czuczman MS, LaCasce AS, Gerecitano JF, Leonard JP, et al. Navitoclax, a targeted high-affinity inhibitor of BCL-2, in lymphoid malignancies: a phase 1 dose-escalation study of safety, pharmacokinetics, pharmacodynamics, and antitumour activity. Lancet Oncol. 2010;11:1149–59. 21094089 10.1016/S1470-2045(10)70261-8 PMC3025495

[37] Kline MP, Rajkumar SV, Timm MM, Kimlinger TK, Haug JL, Lust JA, et al. ABT-737, an inhibitor of Bcl-2 family proteins, is a potent inducer of apoptosis in multiple myeloma cells. Leukemia. 2007;21:1549–60. 17460700 10.1038/sj.leu.2404719

[38] Lalaoui N, Merino D, Giner G, Vaillant F, Chau D, Liu L, et al. Targeting triple-negative breast cancers with the Smac-mimetic birinapant. Cell Death Differ. 2020;27:2768–80. 32341449 10.1038/s41418-020-0541-0 PMC7492458

[39] Czabotar PE, Garcia-Saez AJ. Mechanisms of BCL-2 family proteins in mitochondrial apoptosis. Nat Rev Mol Cell Biol. 2023;24:732–48. 37438560 10.1038/s41580-023-00629-4

[40] Brunelle JK, Letai A. Control of mitochondrial apoptosis by the Bcl-2 family. J Cell Sci. 2009;122:437–41. 19193868 10.1242/jcs.031682 PMC2714431

[41] Elmore S. Apoptosis: a review of programmed cell death. Toxicol Pathol. 2007;35:495–516. 17562483 10.1080/01926230701320337 PMC2117903

[42] Deng J, Paulus A, Fang DD, Manna A, Wang G, Wang H, et al. Lisaftoclax (APG-2575) Is a Novel BCL-2 inhibitor with robust antitumor activity in preclinical models of hematologic malignancy. Clin Cancer Res. 2022;28:5455–68. 36048524 10.1158/1078-0432.CCR-21-4037

[43] Ailawadhi S, Parrondo RD, Dutta N, Han B, Ciccio G, Cherukuri Y et al. AT-101 enhances the antitumor activity of lenalidomide in patients with multiple myeloma. Cancers 2023;15:477. 10.3390/cancers15020477 PMC9857228 36672426

[44] Rath PC, Aggarwal BB. TNF-induced signaling in apoptosis. J Clin Immunol. 1999;19:350–64. 10634209 10.1023/a:1020546615229

[45] Chau BN, Chen TT, Wan YY, DeGregori J, Wang JY. Tumor necrosis factor alpha-induced apoptosis requires p73 and c-ABL activation downstream of RB degradation. Mol Cell Biol. 2004;24:4438–47. 15121862 10.1128/MCB.24.10.4438-4447.2004 PMC400462

[46] Tan X, Martin SJ, Green DR, Wang JY. Degradation of retinoblastoma protein in tumor necrosis factor- and CD95-induced cell death. J Biol Chem. 1997;272:9613–6. 9092486 10.1074/jbc.272.15.9613

[47] Kumarasamy V, Ruiz A, Nambiar R, Witkiewicz AK, Knudsen ES. Chemotherapy impacts on the cellular response to CDK4/6 inhibition: distinct mechanisms of interaction and efficacy in models of pancreatic cancer. Oncogene. 2020;39:1831–45. 31745297 10.1038/s41388-019-1102-1 PMC7047578

